# Impact of Gas Delivery Systems on Imaging Studies of Human Cerebral Blood Flow

**DOI:** 10.1155/2013/694803

**Published:** 2013-12-11

**Authors:** John R. Cain, Laura M. Parkes, Peter Eadsforth, Susan C. Beards, Alan Jackson

**Affiliations:** ^1^Wolfson Molecular Imaging Centre, Cancer and Enabling Sciences, University of Manchester, 23 Palatine Road, Withington, Manchester M20 3LJ, UK; ^2^Biomedical Imaging Institute, University of Manchester, Manchester M20 9PL, UK; ^3^Department of Anaesthetics, Salford Royal Hospital, Stott Lane, Salford, Greater Manchester M6 8HD, UK; ^4^Acute Intensive Care Unit, University Hospital of South Manchester, Southmoor Road, Wythenshawe, Manchester M23 9LT, UK

## Abstract

*Purpose*. To compare a semiopen breathing circuit with a non-rebreathing (Hudson mask) for MRI experiments involving gas delivery. *Methods and Materials*. Cerebral blood flow (CBF) was measured by quantitative phase contrast angiography of the internal carotid and basilar arteries in 18 volunteers (20–31 years). In 8 subjects, gases were delivered via a standard non-rebreathing (Hudson mask). In 10 subjects, gases were delivered using a modified “Mapleson A” semiopen anesthetic gas circuit and mouthpiece. All subjects were given 100% O_2_, medical air, and carbogen gas (95% O_2_ and 5% CO_2_) delivered at 15 L/min in a random order. *Results*. The Hudson mask group showed significant increases in CBF in response to increased FiCO_2_ compared to air (+9.8%). A small nonsignificant reduction in CBF (−2.4%) was seen in response to increased inspired concentrations of oxygen (FiO_2_). The Mapleson A group showed significantly larger changes in CBF in response to both increased inspired concentrations of carbon dioxide (FiCO_2_) (+32.2%, *P* < 0.05) and FiO_2_ (−14.6%, *P* < 0.01). *Conclusions*. The use of an anaesthetic gas delivery circuit avoids entrainment of room air and rebreathing effects that may otherwise adversely affect the experimental results.

## 1. Introduction

Administration of modified gas mixtures is a simple and powerful form of physiological stimulus. Variations in the inspired concentrations of oxygen (FiO_2_) and carbon dioxide (FiCO_2_) in particular are required in a wide range of research studies. Increased FiCO_2_ results in nitric oxide mediated vasodilatation with reduction of cerebral and systemic vascular resistance and secondary increases in cerebral blood volume and cerebral blood flow (CBF) [1]. The carbon dioxide content in fresh air varies between 0.036 and 0.039%. Inhalation of CO_2_ rich mixtures, usually between 2 and 7% in combination with medical air or oxygen (carbogen) is routinely used in combination with MRI to identify areas of reduced cerebrovascular reserve in brains affected by vascular disease [2, 3]. During the past decade, new MRI techniques have also emerged which utilize the effects of increased FiO_2_ on CBF [4, 5], the T1 shortening effect of high FiO_2_ to image oxygen delivery [6, 7], or the changes in haemoglobin saturation to calibrate the Blood-oxygen-level dependent (BOLD) effect [8, 9]. Novel MRI cerebral perfusion techniques are often validated by their ability to detect CBF changes caused by inhaled gas stimuli such as increased FiO_2_ or FiCO_2_ [10].

Many previous studies use nasal cannulae or conventional mask circuit consisting of standard Hudson oxygen masks, with or without a reservoir bag, to deliver gases via standard oxygen tubing from a gas cylinder. In practice, a conventional mask circuit will deliver only 35–40% oxygen, increasing to a maximum of approximately 50% if the oxygen flow is increased above 10 L/min [11]. During a normal inspiration, gas flow reaches a peak of 0.5–1.0 L/min so that a continuous gas flow >60 L/min would be required to ensure that inspired concentrations were equivalent to the concentration delivered. In a conventional mask circuit, the peak inspiratory demand is met by entrainment of air through the holes in the mask or around the seal. Modified gas circuits attempt to reduce this entrainment by use of bags attached to the mask, which fill during expiration to increase the gas delivery rate that is possible during peak inspiration. Delivering oxygen at 10 L per minute to a reservoir bag via 1 meter of 5 mL oxygen tubing will deliver up to 60% oxygen to the patient not the intended 100% [12]. Another major problem with reservoir-based delivery systems is that peak expiratory flow may allow partial filling of the reservoir volume by expired gas, raising the FiCO_2_. Anaesthetic circuits are designed to give greater control over inspired gas concentrations using larger reservoir volumes consisting of both large and low compliance reservoir bags and large volume inflow tubing combined with masks or mouthpieces designed to reduce leakage and entrainment.

Cerebral blood flow (CBF) is highly sensitive to changes in inhaled gas concentrations, particularly CO_2_ and O_2_. CO_2_ is known to cause cerebrovasodilatation and CBF increases of 20–30% are typically seen when breathing 5% carbon dioxide [13]. Very high concentrations of oxygen have been shown to have a vasoconstrictive effect and decrease CBF [14]. CBF decreases from 10 to 15% in healthy subjects breathing 100% oxygen [4]. However, attempts to replicate these finding have been met with variable success [15–17]. Butle et al. found CBF perfusion decreased during 100% O_2_ inhalation using a sealed mask system delivered at 30 L/mim using an Arterial Spin Labeling method [17]. In contrast, Goodwin et al. 2009, who did not detect CBF changes during 100% O_2_ inhalation used an open mask covering the mouth and nose at a flow-rate of 15 L/min using a BOLD method [15].

In this study, we test the hypothesis that gas delivery using a standard face mask and rebreathing bag produces significantly smaller changes in CBF compared to the use of an appropriate anaesthetic gas delivery circuit. This is of considerable importance for human studies using gas mixture administration where underestimation of the inspired gas concentration has the potential to cause major errors of interpretation. For example, there is debate in the literature regarding the effect of both hyperoxia and hypocapnia on CBF, these questions can only be answered by experimentation if there is confidence that the correct concentration of gas is being delivered to the subject.

We have used two different breathing circuits: a conventional mask circuit (Hudson mask) connected to 5 mm oxygen tubing and an anaesthetic design semiopen circuit with a nonresistance bag and low resistance exhaust arm (modified version of the Mapleson A circuit). We measured resulting changes in cerebral blood flow in normal young volunteers using phase-contrast angiography MRI.

## 2. Methods and Materials

### 2.1. Subjects

18 healthy normal volunteers (6 females and 12 males) who had given written informed consent were included in the study. Exclusion criteria included smokers, asthmatics, individuals with significant heart or lung disease, and contraindications to MRI. The age range was 20–31 years (mean 25.1 years).

### 2.2. Ethics Statement

Approval for the study was granted by the Health Research Authority, National Research Ethics Service, Greater Manchester Central Research Ethics Committee (reference numbers 05/Q1410/124 and 06/Q1410/66), and all subjects were given a minimum of 24 hours to consider inclusion.

### 2.3. Gas Circuits

MR imaging was performed using a 3.0 T Philips Achieva system (Philips Medical Systems, Best, The Netherlands) using an 8 channel head coil. The subjects were randomly allocated to two groups. In 8 subjects, gas mixtures were delivered via 3 meters of 5 mm diameter oxygen tubing and a standard reservoir bag mask (Hudson mask; Henleys Medical, Welwyn Garden City, Hertfordshire, UK). In the remaining 10 subjects, gases were delivered using a modified “Mapleson A” semi-open anaesthetic gas circuit and a mouthpiece (subjects also wore a nose clip) [18]. Previous groups have described increased motion artefact during carbon dioxide inhalation; therefore, care was taken to ensure the subjects heads were secured in position as tightly as practicable [19].

**Figure 1:**
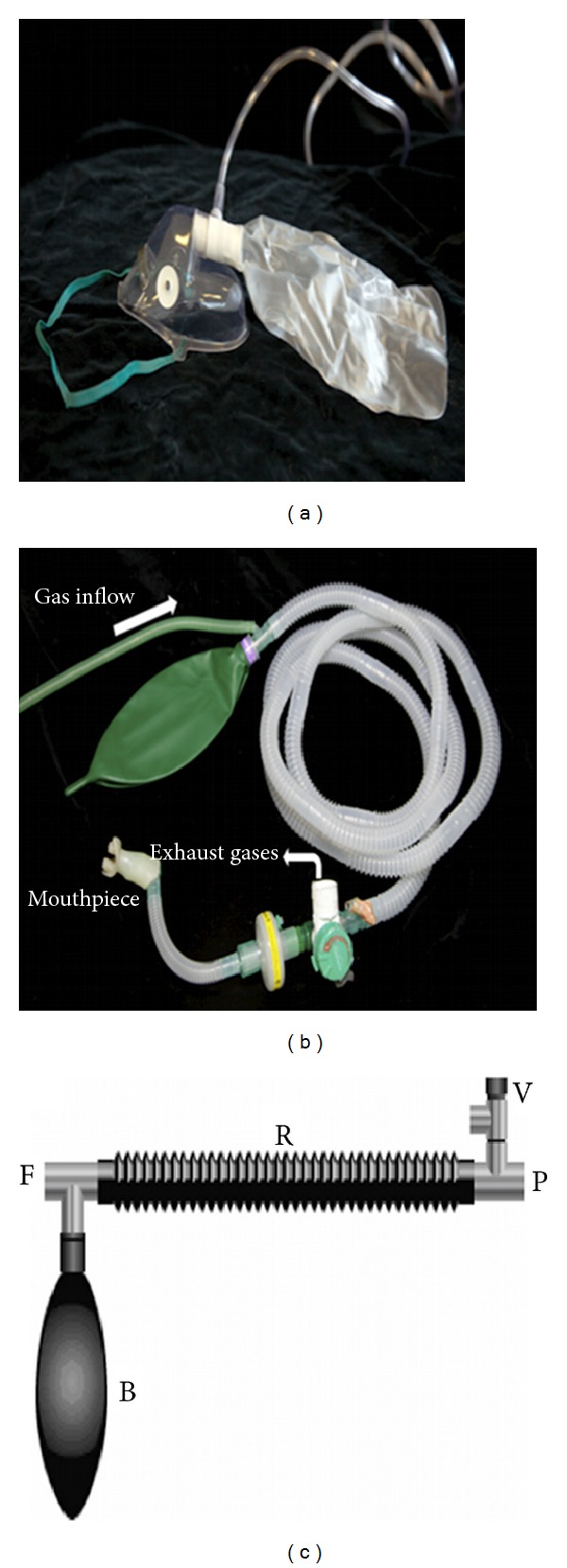
(a) Hudson mask with oxygen tubing. (b) Mapleson A semi-open circuit with mouthpiece (exhaust tubing removed for clarity). (c) Schematic diagram of Mapleson A circuit: 3 way T-tube connected to the fresh gas outlet (F), a breathing bag (B), and a reservoir tube (R). Reservoir tube is connected to the patient (P) and a spring-loaded expiratory valve (V) (image courtesy of Anaesthesia UK (http://www.frca.co.uk/).

The Mapleson A circuit was modified to maintain inspired gas concentrations as close to the delivered concentration as possible by reducing incidental gas entrainment by use of a mouthpiece and nose clip and by provision of a large inflow reservoir bag (Figure 1). The exhaled gases were expelled outside the scan room by a wide bore “scavenging” exhaust system connected to the circuits' expiratory valve. The presence of rebreathing was monitored using a capnograph to continuously monitor End-tidal CO_2_ (ETCO_2_)_._ Although arterial sampling of partial pressure CO_2_ (PaCO_2_) would be more sensitive, arterial line insertion was felt inappropriate in normal volunteers.

All subjects were given the following gases delivered in a random order, each for approximately 15 minutes duration: 100% O_2_, medical air, and carbogen gas (95% O_2_, 5% CO_2_). All were delivered at a flow rate of 15 L/min.

During the MRI scan, the patients pulse and oxygen saturation were continuously measured using an MRI compatible pulse oximeter using a Precess 3160 MRI patient monitoring system (Invivo, Florida, USA). End-tidal CO_2_ (ETCO_2_) was estimated non-invasively using a capnograph (Invivo, FL, USA) sampling from within the mask. Intermittent blood pressure was measured every 3 minutes via an automated cuff.

Imaging consisted of the following sequences.

- *2D axial T*_2_ *-weighted turbo spin echo* with full brain coverage: echo time (TE) 80 ms, repetition time (TR) 3000 ms, flip angle 90°, matrix 512 × 512, pixel size 1.17 × 1.17 mm, slice thickness 4 mm, slice gap 5 mm, and slices 24. This sequence was to ensure the subject did not have any incidental pathology which could affect their cerebrovascular reactivity; a neuroradiologist reviewed the images.
- *Phase contrast cerebral angiographic survey*: both coronal and sagittal planes: TE 6.3 ms, TR 20 ms, flip angle 15°, number of averages 2, matrix 256 × 128, pixial size 0.97 × 0.97 mm, and slice thickness 80 mm. The angiographic survey was used to evaluate cerebral circulation and identify internal carotid arteries and basilar artery.
- *2D Quantitative Phase Contrast Angiography* (PCA): acquisition was collected using axial 2D cine phase-contrast images. ECG cardiac gating was used to cover the entire cardiac cycle. 256 acquisitions were continuously acquired, during image reconstruction, the recorded ECG trigger signals are used to retrospectively assign the data to the different positions in the cardiac cycle to create 16 phase images over the cardiac cycle [20]. The imaging parameters were TE 4.43 ms, TR dependant on heart rate ranging between 7.4–14.1 ms, flip angle 10°, number of averages 3, matrix 256 × 256, pixel size 1.17 × 1.17 mm, slice thickness 6 mm, and velocity encoding 200 cm/s. For each subject, a single 2D PCA slice was collected at the level of the skull base, perpendicular to the internal carotid and basilar arteries. The acquisition was repeated throughout the administration of the gas challenges [21].

### 2.4. Analysis

PCA results were analysed using Segment flow software [22]. The MRI PCA sequence generates phase difference image from which the distance the blood has moved in the imaging time through the vessel can be measured (units: cm). The stroke distance is measured 16 times over the cardiac cycle and averaged to give a stroke distance average over the heartbeat (units: cm per heartbeat). This number is multiplied by the measured heart rate to give flow velocity (units: cm min^−1^). Flow velocity is multiplied by the cross sectional area of the vessels (information from the magnitude information of the PCA MRI scan) to give the flow volume for the vessel (mL min^−1^). The flow volume of the two internal carotid and basilar arteries is added to give total CBF [18].

The group CBF results were compared both between each gas on each circuit and between the absolute flow values across circuits on the same gas using *t*-tests, (significance assumed at *P* < 0.05).

## 3. Results

All subjects tolerated the experiment and no significant adverse effects were reported in either group. Subjects subjectively noted an increase in anxiety and respiration rate during carbogen inhalation and this was more pronounced with the Mapleson A circuit. There was no appreciable increase in image artefacts identified due to the motion between each of the gases with either circuit.

In both groups, the heart rate (62 mean) mean arterial blood pressure (MAP) (79 mmHg mean) and blood oxygen saturation (97–100%) remained constant throughout gas administration.

Although there was a slight increase in respiratory rate during carbogen inhalation, the ETCO_2_ showed no significant changes during either oxygen or carbogen inhalation compared to air using the modified Mapleson A circuit (see Table 1). Measurements of ETCO_2_ proved to be unreliable since monitoring was performed within the mask, where the sampling is contaminated by inflowing gas, measurements were artificially low on air and 100% oxygen and high during carbogen administration.

**Figure 2:**
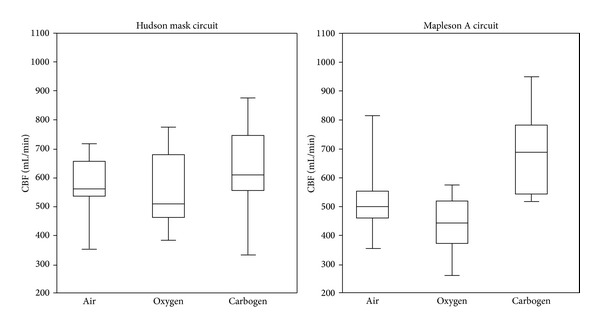
Box plot (median, quartiles) of CBF results under each gas. Hudson Mask circuit = reservoir bag mask, Mapleson A = semi-open anaesthetic circuit.

Changes in CBF are shown in Table 2 and Figure 2. The Hudson mask group showed significant increases in CBF in response to increased FiCO_2_ compared to air (+9.8%). A small reduction in CBF (−2.4%) was seen in response to increased FiO_2_ but this failed to reach significance. The Mapleson A group showed significantly larger changes in CBF in response to both increased FiCO_2_ (+32.2%, *P* < 0.05) and FiO_2_ (−14.6%, *P* < 0.01) both of which were statistically significant.

## 4. Discussion

This study demonstrates that in order to reliably administer modified gas mixtures, the use of a correctly designed breathing system gas delivery circuit is important. This has direct relevance for a large number of imaging studies, which rely on physiological manipulation of inspired gas content. The principle differences between the circuits used in this paper are (1) the improved seal obtained by use of a mouthpiece and nose clip rather than a face mask, providing no significant means of entraining room air into the circuit, (2) the use of a large resistance-free reservoir bag to provide adequate reserve for peak inspiratory flow rates, and (3) effective removal of waste gases by an exhaust system.

The ideal gas delivery circuit for controlling the oxygen alveolar concentration in an MRI scanner would be a closed Bain circuit in an intubated patent [23]. This obviously is not feasible for most research MRI scans and a compromise between optimum gas delivery and participant comfort must be found. We have employed a version of a standard anaesthetic circuit known as the Mapleson A. The Mapleson A circuit allows some rebreathing of expired gases which pass into the inflow tube during early expiration. The combination of expiration and the pressure of the inflowing gas mixture then opens the exhaust valve so that gas from late expiration is exhausted. The reason for this design is to allow the reuse of exhaled dead space gas, which retains the same gas concentrations as inflowing gases. Rebreathing of alveolar gas can be prevented if the fresh gas flow is equal to or greater than 0.7 × the patients minute volume. In a study of 16 patients, this ranged from 3.1 to 4.6 L/min [24] so that with a high inflow rate, such as the 15 L/min used in this study, no rebreathing will occur.

There are a number of considerations when using gas circuits in an MRI environment. Safety of participants is of paramount importance. Standard Hudson mask devices and nasal cannulae expel excess fresh gas and expired gas directly into the scanner bore. This has the potential for high concentrations of flammable gas to build up, presenting a potential fire risk. The use of Mapleson A or Bain circuit with a “scavenging” exhaust system in which expired gases are expelled outside the scan room removes this risk.

In conclusion, MRI experiments to study physiological or pathological processes involved in the control of regional blood flow using methodologies such as Arterial Spin Labelling often use inhalation of modified gas mixtures to evoke perfusion changes. This study highlights the importance of choosing the correct gas circuit design in such experiments alongside other factors of experimental design.

## Figures and Tables

**Table 1:** End tidal CO_2_ and respiratory rate under each gas using both circuits.

Gas	Hudson mask circuit	Mapleson A circuit	
	Respiratory rate RPM	Respiratory rate RPM	ETCO_2_ mmHg
Medical air	14	11	38
Oxygen	11.8	13.7	33
Carbogen	13.2	16.8	42

**Table 2:** CBF results are shown for both circuits and each gas.

GAS	Hudson mask circuit CBF (mL min^−1^)	(SD)	Mapleson A circuit CBF (mL min^−1^)	(SD)
Medical air	571	108	515.6	120
Oxygen	557 (−2.4%)	142	440.2 (−14.6%)*	89
Carbogen	624 (+9.8%)*	169	681.7 (+32.2%)***	148

Figures are mean values and standard deviations are also given. Figures in brackets represent the percentage change in mean value compared to air. Significance values are calculated using paired *t*-test in comparison with medical air on the same breathing circuit *P < 0.05, **P < 0.01, ***P < 0.001.
